# Optimisation of geometric aspect ratio of thin film transistors for low-cost flexible CMOS inverters and its practical implementation

**DOI:** 10.1038/s41598-022-19989-6

**Published:** 2022-09-27

**Authors:** N. C. A. van Fraassen, K. M. Niang, J. D. Parish, A. L. Johnson, A. J. Flewitt

**Affiliations:** 1 Electrical Engineering Division, Engineering Department, Cambridge University, Cambridge, CB3 0FA UK; 2 Department of Chemistry, University of Bath, Bath, BA2 7AX UK

**Keywords:** Electrical and electronic engineering, Electronic and spintronic devices

## Abstract

A low-cost, flexible processor is essential to realise affordable flexible electronic systems and transform everyday objects into smart-objects. Thin film transistors (TFTs) based on metal-oxides (or organics) are ideal candidates as they can be manufactured at low processing temperatures and low-cost per unit area, unlike traditional silicon devices. The development of complementary metal–oxide–semiconductor (CMOS) technology based on these materials remains challenging due to differences in performance between n- and p-type TFTs. Existing geometric rules typically compensate the lower mobility of the metal-oxide p-type TFT by scaling up the width-to-length (*W/L*) ratio but fail to take into account the significant off-state leakage current. Here we propose the concept of an optimal geometric aspect ratio which maximises the inverter efficiency represented by the average switching current divided by the static currents. This universal method is especially useful for the design of low-power CMOS inverters based on metal-oxides, where the large off-current of the p-type TFT dominates the static power consumption of the inverter. We model the inverter efficiency and noise margins of metal-oxide CMOS inverters with different geometric aspect ratios and compare the performance to different inverter configurations. The modelling results are verified experimentally by fabricating CMOS inverter configurations consisting of n-type indium-silicon-oxide (ISO) TFTs and p-type tin monoxide (SnO) TFTs. Notably, our results show that reducing *W/L* of metal-oxide p-type TFTs increases the inverter efficiency while reducing the area compared to simply scaling up *W/L* inversely with mobility. We anticipate this work provides a straightforward method to geometrically optimise flexible CMOS inverters, which will remain relevant even as the performance of TFTs continues to evolve.

## Introduction

Flexible electronic devices are fabricated on substrates such as paper, polymer and metal foil^1^. Metal-oxides, organics and amorphous silicon are commonly used active materials. Compared to traditional silicon devices, they offer a number of advantages including thinness, conformability and low manufacturing costs. Mature low-cost, thin, flexible and conformable devices have been succesfully developed. These include sensors^2^, memories^3^, batteries^4^, light-emitting diodes, energy harvesters^5^, near-field communication/radio frequency identification^6^ and printed circuitry such as antennas; essential electronic components to build any smart integrated electronic device. A low-cost flexible microprocessor employing CMOS technology is yet to be realised. Silicon processors are unsuitable as they are unlikely to reach a price point at which everyday items, such as bottles, food packaging, and wearables, can be turned into smart-objects. Therefore, there is a strong interest in low-power circuit designs and larger-scale integration of flexible thin film transistors (TFTs). Processors have been fabricated using low-temperature poly-silicon (LTPS) TFTs^7^ but high manufacturing costs and poor scalability of this technique make it unsuitable for high-volume, low-cost, flexible integrated smart systems^8^. Organics are also actively researched and excellent low-voltage CMOS inverters have been reported^9–11^. However, their use is limited to low-end backplane and circuit applications due to lower mobility, stability, uniformity and limited scalability^8^. Metal-oxides are arguably the most promising due to their high mobility, excellent spatial uniformity and scalability. A flexible processing engine fabricated with 0.8 μm n-type metal-oxide TFT technology has recently been reported^12^. It contains ~ 1000 gates (resistive load logic) and its gate-density is 45 times higher than previous metal-oxide processors^3,6,12–14^. The same authors have since fabricated a flexible 32-bit processor consisting of 18,334 gates^14^. Earlier this year, the same TFT technology was used to fabricate a flexible microprocessor using pseudo-CMOS logic^15^. While these works show the potential of metal-oxide processors, they also highlight its main shortcoming; only n-type TFTs were used since there is currently considered to be no compatible p-type material. Any further increase in gates requires CMOS technology as the static power consumption, *P*_stat_, of unipolar technology becomes unfeasibly high^14,15^.

CMOS technology, which combines n- and p-type TFTs, benefits from low power consumption, high circuit integration density, high logic output, and high noise margins^16^. The development of thin film CMOS is therefore vital for low-cost flexible processors. Ideally, the output characteristics of n- and p-type TFTs in complementary inverters are perfectly matched. Indeed, the success of silicon CMOS is partly due to excellent control of n- and p-type MOSFET characteristics. In contrast the use of oxides and organics is challenging due to poor performance of oxide p-type and n-type organic TFTs relative to silicon MOSFETs.

Development of p-type oxide TFTs has been hampered by the low mobility and current-switching-ratio (*I*_on_/*I*_off_) caused by the high off-state current, *I*_off_, that is typically observed^17,18^. Cuprous oxide (Cu_2_O) and tin monoxide (SnO) TFTs demonstrate promising results but mobilities are generally limited to ~ 1 cm^2^/Vs^18,19^. Both these materials, but primarily SnO, have been used in all-oxide CMOS inverters^19,20^. A high geometric aspect ratio, (*W/L*)_p_/(*W/L*)_n_, is normally used to increase the maximum output current, *I*_max_, of the p-type TFT to match the n-type TFT using the same design rule that is applied to silicon CMOS.

**Figure 1:**
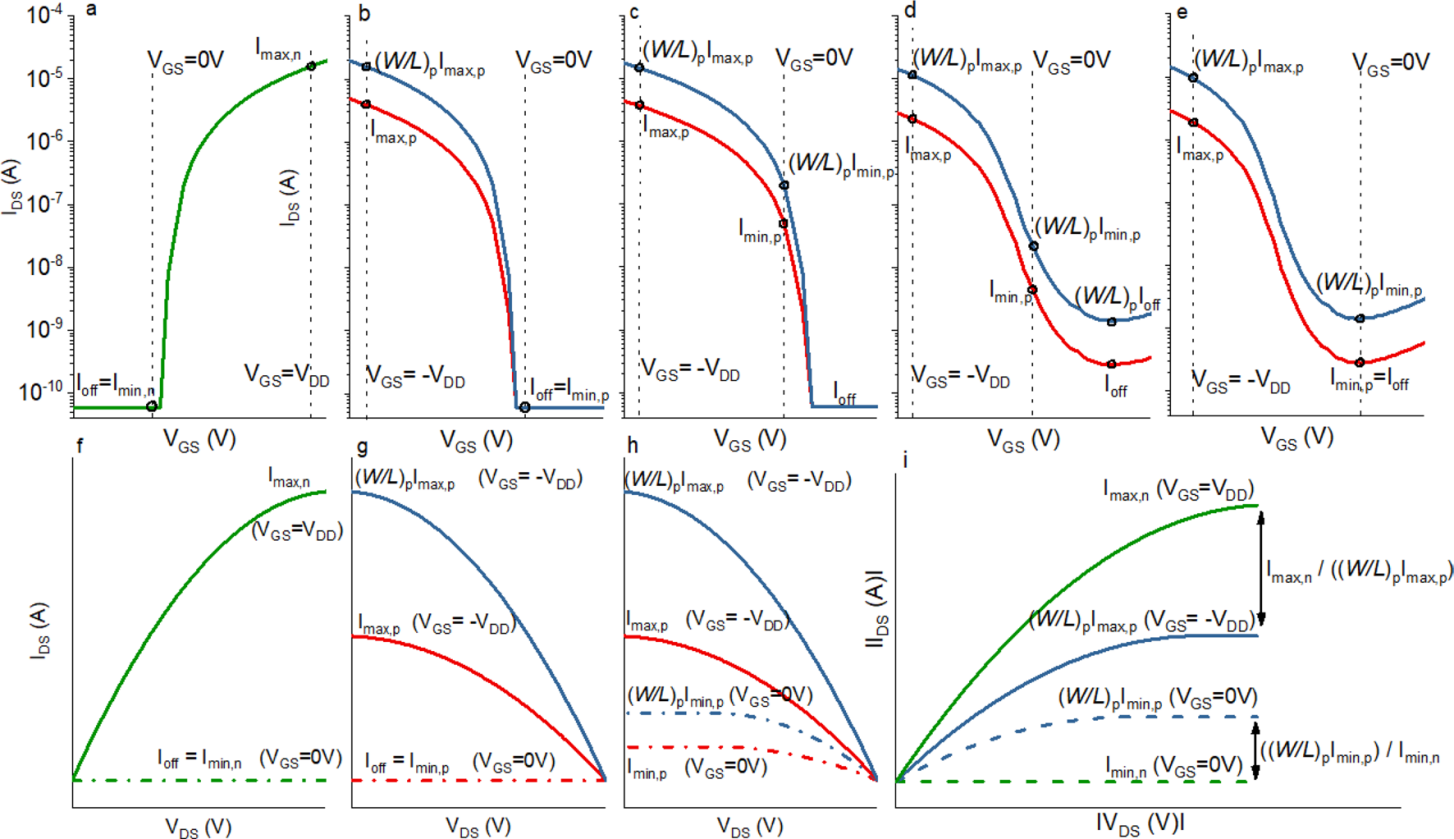
Transfer characteristics and output characteristics of: (**a**,**f**) ‘ideal silicon’ n-type transistor with *V*_on_ ~ 0 V and complete channel depletion; *I*_off_ = *I*_min,n_ does not scale with (*W/L*)_n_. (**b**,**g**) p-type transistor with *V*_on_ ~ 0 V and complete channel depletion; to match the higher mobility of the n-type, *I*_max,p_ is scaled by (*W/L*)_p,_ *I*_off_ = *I*_min,p_ does not scale with (*W/L*)_p_. (**c**,**h**) p-type transistor with *V*_on_ > 0 V and complete channel depletion; at *V*_GS_ = 0 V the transistor is partly turned on and both *I*_max,p_ and *I*_min,p_ scale with (*W/L*)_p_. (**d**,**e**,**h**) ‘oxide’ p-type transistor without complete channel depletion; both *I*_off_ and *I*_min,p_ scale with (*W/L*)_p_. (**i**) Maximum and minimum output currents for n- (green) and p-type transistors, and the ratio between them after scaling with (*W/L*)_p_.

For silicon CMOS this method results in an excellent match of the n- and p-type output characteristics provided the threshold voltages (*V*_th_) of the n- and p-type transistor are approximately equal and opposite in sign (ideally close to 0 V). Moreover, the transistors should fit the standard MOSFET equations well with a constant saturation mobility. As a result, the propagation delays of the low-to-high and high-to-low transitions are roughly equal and the switching voltage is approximately *V*_DD_/2, maximising noise margins. In the Supplementary Information, the static states of the CMOS inverter and dynamic low-to-high and high-to-low transitions are illustrated, along with the terminology for the different performance variables used in this work (Fig. S1). Ideally, both the n- and p-type transistors exhibit complete channel depletion in the off-state and have a turn-on voltage, *V*_on_, of ~ 0 V. In this case, the minimum output current at *V*_GS_ = 0 V, *I*_min_, and *I*_off_ both remain constant as the width-to-length ratio (*W/L*) (or *V*_DS_) is increased. Since the static current of the p-type device equals *I*_min_, increasing (*W/L*)_p_ (or *V*_DS_) does not affect *P*_stat_ of the inverter. This represents the ideal (‘silicon’) scenario (Fig. 1a,b,f,g). Note there are subthreshold models for Si transistors showing the off-current roughly scales with *W/L* and therefore *P*_stat_ of the inverter does increase with *W/L*^21^. However, for Si CMOS inverters the effect is negligible as the overall power consumption is dominated by the dynamic power (due to the higher operation frequency and low off-current).

Simply scaling *W/L* inversely with mobility does not work well for (flexible) TFTs based on metal-oxides and organics as large differences (in mobility, subthreshold swing, *I*_on_, *I*_off_, *V*_th_) remain between the performance of n- and p-type TFTs. It is therefore surprising to see that this method is widely used for TFT-based CMOS inverters. As reported for Cu_2_O^17^, we observe in SnO that *I*_off_ scales with *V*_DS_ and (*W/L*)_p_. This dependence is likely due to accumulation of electrons in the off-state, which decreases channel resistance^17^. This suggests a common mechanism for p-type oxides. Therefore, increasing (*W/L*)_p_ also increases *I*_off_ and *I*_min_ (Fig. 1d,e,h). Considering *I*_off_ of metal-oxide p-type TFTs is typically at least tenfold higher than for its n-type counterpart, simply scaling up (*W/L*)_p_ so that *I*_max,n_ = (*W/L*)_p_*I*_max,p_ creates an even greater mismatch between the off-currents and directly increases *P*_stat_ of the inverter. A similar effect can be observed for p-type (n-type for organics case, referred to in brackets from here onwards) transistors where *V*_on_ is considerably above (below) 0 V. The transistor is now partly turned on at *V*_GS_ = 0 V and *I*_min_ also scales with *V*_DS_ and *W/L* (Fig. 1c,h). This can be observed in many transistors, including n-type organic and p-type oxide TFTs, where precise control of characteristics remains challenging. The dependence of *I*_min_ on *W/L* is likely to remain an issue even as the performance of TFTs improves. Moreover, new transistor technologies may emerge in the future with similar characteristics.

In this work, we experimentally investigate the effects of changing *W/L* of a p-type SnO TFT on the voltage transfer characteristics (VTC) and current transfer characteristics (CTC) of all-oxide CMOS inverters. We fabricated n-type amorphous indium-silicon-oxide (a-ISO) TFTs and p-type SnO TFTs to combine them into all-oxide CMOS inverters. A model was developed to verify CMOS performance and compare it to the ideally matched case, as well as a unipolar resistive load inverter. We define inverter efficiency, *I*_p_/*I*_stat_, as the average switching current (*I*_p_) divided by the sum of the static currents (*I*_stat_). We show that reducing *W/L* of oxide p-type TFTs increases *I*_p_/*I*_stat_ compared to simply scaling up *W/L* inversely with mobility, while reducing the area.

Finally, we propose the concept of an optimal geometric aspect ratio which is universally applicable to silicon, metal-oxide and organic complementary inverters. This ratio determines the *W/L* of the p-type (n-type) transistor that best matches the maximum and minimum output currents of both n- and p-type TFTs equally so that inverter efficiency is maximised. This is critical to reduce the static power consumption (*P*_stat_) and enable large-scale integration of metal-oxide TFTs. We estimate that by using the approach developed in this work it is possible to reduce *P*_stat_ by a factor > 100 and increase *I*_p_/*I*_stat_ by a factor > 100, compared to unipolar resistive technology, based on the current performance of p-type SnO TFTs, thus enabling a further increase in gate density.

## Characteristics of n-type a-ISO and p-type SnO TFTs

**Figure 2:**
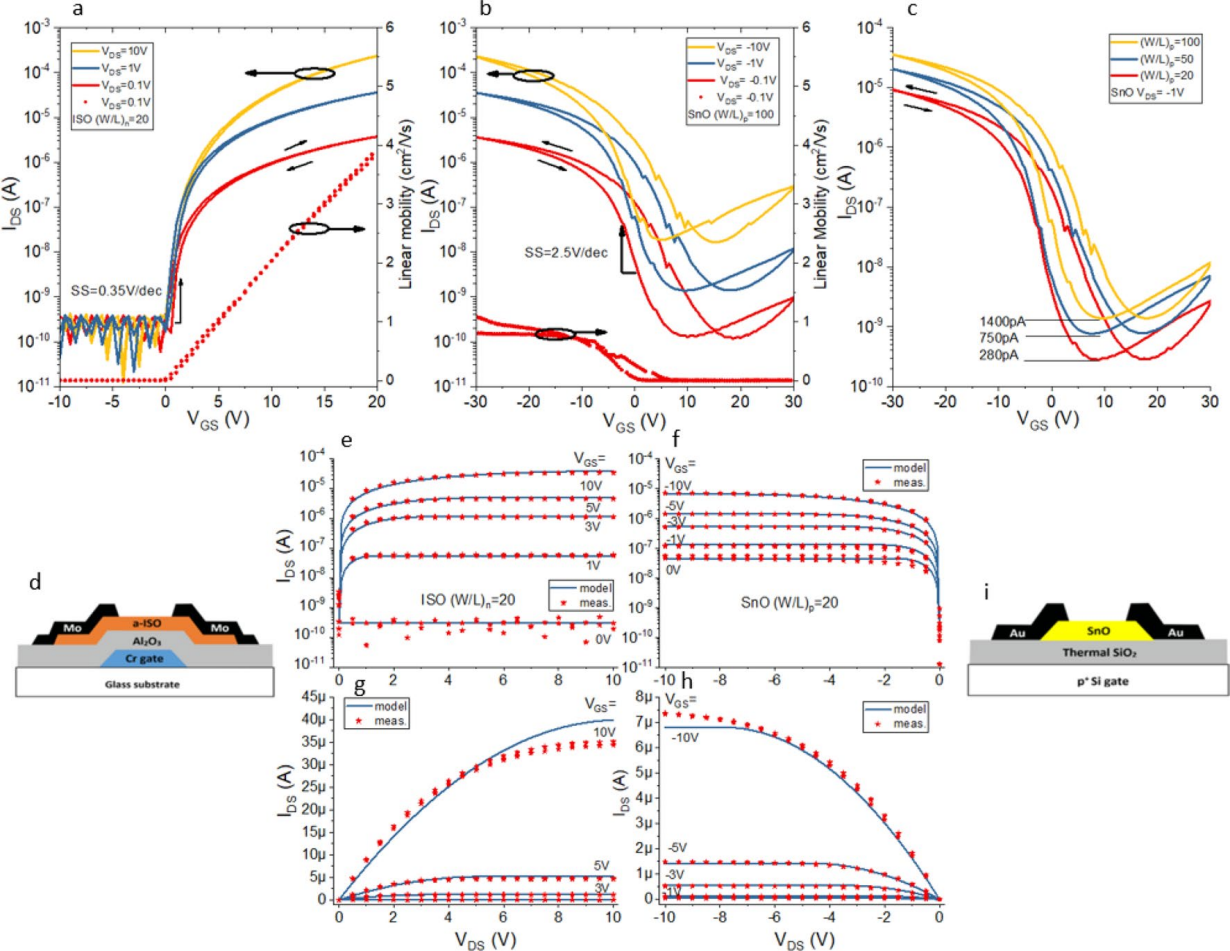
Experimentally measured transfer characteristics and linear mobility of (**a**) a-ISO TFT and (**b**) SnO TFT. Transfer characteristics of SnO TFT with different *W/L* ratios (**c**). Output characteristics (red) and modelled curves (blue) of the a-ISO TFT and the SnO TFT (**b**,**d**) in log (**e**,**f**) and linear scales (**g**,**h**). Schematic cross section of (**d**) the a-ISO TFT and (**i**) SnO TFT.

Figure 2d,i show schematics of the a-ISO and SnO TFTs (fabrication details provided in “Methods”). The a-ISO TFT (*W/L* = 20) has an ideal *V*_on_ of ~ 0 V and *I*_off_ of ~ 400 pA as shown in Fig. 2a. *I*_off_ is independent of *V*_DS_ which demonstrates complete channel depletion. The subthreshold swing (*SS*) is 0.35 V/dec, the *I*_on_/*I*_off_ is ~ 10^6^ for *V*_DS_ = 10 V, and the threshold voltage (*V*_th_) is 0.2 V. The mobility increases linearly and reaches 4 cm^2^/Vs at *V*_GS_ = 20 V. For the SnO TFT (Fig. 2b,c), *V*_on_ is ~ 8 V and *I*_off_ ranges from 200 pA to 20 nA as *V*_DS_ varies from − 0.1 to − 10 V (for *W/L* = 100) indicating the difficulty to turn off the device. This scaling of *I*_off_ with *V*_DS_ and *W/L* is commonly observed for p-type oxides like SnO and Cu_2_O^17,22–25^. The SnO transfer characteristics in Fig. 2c exhibit similar dependence; for *W/L* ratios of 20, 50 and 100, *I*_off_ is 280, 750 and 1400 pA respectively. The parameters of the SnO TFT are as follows; *SS* = 2.5 V/dec, the *I*_on_/*I*_off_ is ~ 2 × 10^4^ for *V*_GS_ = [− 10 V, 30 V], and *V*_th_ = 2.3 V. The mobility increases linearly up to *V*_GS_ = − 10 V and then saturates at ~ 1 cm^2^/Vs, as shown in Fig. 2b. Hysteresis is a common problem for SnO TFTs and is likely caused by the high trap state density near the interface between the SnO layer and the SiO_2_ insulator. It has been shown that (alumina) interfacial layers can help to reduce the high trap state density^26^.

The output characteristics of both TFTs exhibit a clear linear and saturation region as shown in Fig. 2g,h (*W/L* = 20 for both TFTs). The saturation current of the a-ISO TFT ranges from *I*_min,n_ = 300 pA (*V*_GS_ = 0 V) to *I*_max,n_ = 35 µA (*V*_GS_ = 10 V). The range of the SnO TFT ((*W/L*)_p_ = 20) is smaller and varies from *I*_min,p_ = 60 nA to *I*_max,p_ = 7 µA. In a CMOS inverter, the output characteristics of the n- and p-type transistors are ideally matched exactly, and usually (*W/L*)_p_ is increased to match the higher mobility and on-current of the n-type device. This can be achieved by setting (*W/L*)_p_ = 100. In this case the output currents align more closely for *V*_GS_ = [3, 5, 10 V] but the gap increases for *V*_GS_ = [0, 1 V] since *I*_off_ of the p-type device scales with (*W/L*)_p_. This raises the question whether increasing (*W/L*)_p_ actually improves the inverter performance.

## Modelling of inverters

A model for the output characteristics was developed based on the standard MOSFET equations modified by a pre-factor, $\alpha \left({V}_{GS}-{V}_{\mathrm{th},\mathrm{n}}\right)$, where *V*_th,n_ is the threshold of the n-type TFT. This takes into account the linear dependence of mobility on *V*_GS_, as shown in Fig. 2a,b. The equations for the a-ISO and SnO TFTs in the linear regime respectively are:

1 $${I}_{\mathrm{DS},\mathrm{n}}={\left(\frac{W}{L}\right)}_{n}{C}_{\mathrm{ox},\mathrm{n}}\alpha \left({V}_{\mathrm{GS}}-{V}_{\mathrm{th},\mathrm{n}}\right)\left(\left({V}_{\mathrm{GS}}-{V}_{\mathrm{th},\mathrm{n}}\right){V}_{\mathrm{DS}}-\beta {{V}_{\mathrm{DS}}}^{2}\right)+{I}_{\mathrm{OFF},\mathrm{n}},$$

2 $${I}_{\mathrm{DS},\mathrm{p}}={\left(\frac{W}{L}\right)}_{p}\left({C}_{\mathrm{ox},\mathrm{p}}\gamma \left({V}_{\mathrm{GS}}-{V}_{\mathrm{th},\mathrm{p}}\right)\left(\left({V}_{\mathrm{GS}}-{V}_{\mathrm{th},\mathrm{p}}\right){V}_{DS}-\delta {{V}_{\mathrm{DS}}}^{2}\right)+{I}_{\mathrm{OFF},\mathrm{p}}\right).$$

**Figure 3:**
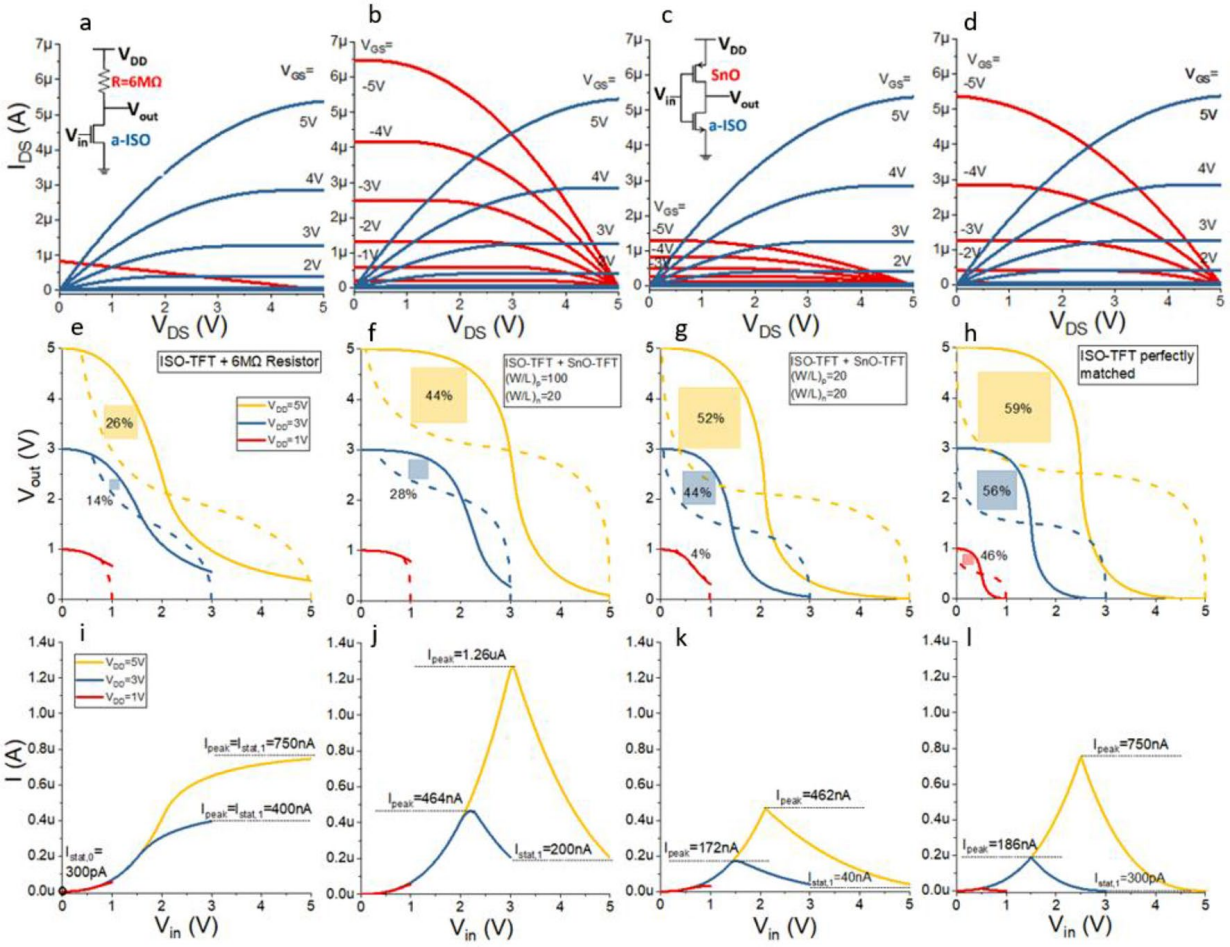
Output characteristics of ISO TFT *(W/L)*_n_ = 20 (blue) with (**a**) 6MΩ resistor, (**b**) SnO *(W/L)*_p_ = 100, (**c**) SnO *(W/L)*_p_ = 20, (**d**) perfectly matched p-type TFT. (**e**–**h**) Corresponding inverter voltage transfer characteristics, MEC noise margins are represented by the largest square between the transfer curves. (**i**–**l**) Inverter current transfer characteristics.

In the saturation regime, *I*_DS,n_ and *I*_DS,p_ equal the maximum value when $\frac{\partial {I}_{\mathrm{DS}}}{\partial {V}_{\mathrm{DS}}}=0$ (further details in Supplementary Information). Figure 2e–h show the model closely fits the measured data. The logarithmic graphs in Fig. 2e,f show a good match for lower *V*_GS_ and especially for *I*_min_, which represents the static off-current of the CMOS inverter when *V*_in_ = *V*_GS_ is low (0 V) and high (*V*_DD_) for the n- and p-type devices, respectively. Figure 2g,h demonstrate a good fit on a linear scale at higher *V*_GS_. We created a MATLAB model of an inverter in which the n-type a-ISO TFT functions as the pull-down network with a range of different loads. The model simulates the VTC and CTC based on the intersection points of the output characteristics. Figure 3 shows the simulation results of four different configurations.

The configuration in Fig. 3a,e,i contains a 6 MΩ load and is similar to unipolar resistive inverters currently used in several flexible processors^12–14^. The VTC show a limited output swing since *V*_out_ > 0.5 V. The Maximum Equal Criteria (MEC) noise margin (NM) is 26% of the maximum value of *V*_DD_/2 for *V*_DD_ = 5 V. The output swing and NMs decline drastically as *V*_DD_ is reduced to 3 V and 1 V. The CTC shows the static current at *V*_in_ = 0 V (*I*_stat,0_) is negligible (*I*_stat,0_ = *I*_min,n_ = 300 pA). The resistor current increases, reaching its peak (*I*_peak_) when *V*_in_ = *V*_DD_. Therefore, the static current at *V*_in_ = *V*_DD_ (*I*_stat,1_) is 400 nA and 750 nA for *V*_DD_ = 3 V and *V*_DD_ = 5 V respectively, which is > 1000-fold greater than *I*_stat,0_.

In Fig. 3d,h,l the resistor is replaced with a theoretically ideal p-type TFT with the same output characteristics as the n-type a-ISO TFT to form a complementary inverter. This represents an ideal setup for comparison as metal-oxide p-type TFTs with this performance do not currently exist. The CMOS inverter characteristics are excellent; even at *V*_DD_ = 1 V it achieves a rail-to-rail swing and NM = 46%. Most importantly the p-type TFT turns off when *V*_in_ = *V*_DD_, meaning *I*_stat,1_ = *I*_min,p_ = 400 pA. *I*_peak_ is still ~ 750 nA when *V*_in_ = *V*_DD_/2 but both of the static currents are only 400 pA, reducing *P*_stat_ by > 1000-fold. This advantage of CMOS operation is essential for high-density circuits.

VTCs, NMs and gain are widely reported for inverters but provide little information about switching speed and (static) power consumption (i.e. from this information alone it is impossible to tell whether an inverter is an ‘efficient’ CMOS inverter or a resistive load inverter). Here we define a dimensionless inverter efficiency, *I*_p_/*I*_stat_, as the average switching current divided by the sum of the static currents, (*I*_stat_ = *I*_stat,0_ + *I*_stat,1_). The average switching current, *I*_p_, is defined such that it scales with the switching frequency (*f*). *f* scales with the inverse of the overall propagation delay (*t*_p_), where *t*_p_ equals the sum of the high-to-low (*t*_pHL_) and low-to-high (*t*_pLH_) transitions. For constant *V*_DD_, the time taken to discharge and charge a constant load capacitance (*C*_L_) scales with the inverse of the maximum discharge (*I*_max,HL_) and charge current (*I*_max,LH_) respectively (see Eq. (3) and Fig. S1). Therefore, *f* scales with *I*_max,HL_*I*_max,LH_/(*I*_max,HL_ + *I*_max,LH_) which we define as *I*_p_.

3 $$f\propto \frac{1}{{t}_{p}} =\frac{1}{{t}_{pHL}+{t}_{pLH}} \propto \frac{1}{\frac{{C}_{L}{V}_{DD}}{{I}_{max,HL}}+\frac{{C}_{L}{V}_{DD}}{{I}_{max,LH}}} \propto \frac{{I}_{max,HL}{I}_{max,LH}}{{I}_{max,HL}+{I}_{max,LH}}={I}_{p}$$

Note in reality the shape of *I*_max,HL_ and *I*_max,LH_ (as a function of the voltage across *C*_L_) also affects *t*_pHL_ and *t*_pLH_ but we ignore this as a second order effect. This leads to the dimensionless definition for the inverter efficiency in Eq. (4). This is an excellent performance parameter as it leverages switching speed over static power and provides a way to quantify this.

4 $$inverter\,\, efficiency{:} \frac{{I}_{p}}{{I}_{stat}} =\frac{\frac{{I}_{max,HL}{I}_{max,LH}}{{I}_{max,HL}+{I}_{max,LH}}}{{I}_{stat,0}+{I}_{stat,1}}$$

For the basic n-type unipolar resistive load inverter *I*_max,HL_ = *I*_max,n_, *I*_stat,0_ = *I*_min,n_, and *I*_max,LH_ = *I*_stat,1_ = *I*_max,R_ where *I*_max,R_ is the peak current through the resistor. This results in an upper bound of *I*_p_/*I*_stat_ < 1 (Eq. (5)) since *I*_min,n_ is generally negligible compared to *I*_max,R_ and represents a ‘worst-case scenario’. For the inverter in Fig. 3a,e,i *I*_p_/*I*_stat_ = 0.86.

5 $$n{\text{-}}type\,\, resistive\,\, load{:}\, \frac{{I}_{p}}{{I}_{stat}} =\frac{\frac{{I}_{max,n}{I}_{max,R}}{{I}_{max,n}+{I}_{max,R}}}{{I}_{min,n}+{I}_{max,R}}\approx \frac{{I}_{max,n}}{{I}_{max,n}+{I}_{max,R}}<1$$

For the CMOS inverter, *I*_max,HL_ = *I*_max,n_, *I*_max,LH_ = *I*_max,p_, *I*_stat,0_ = *I*_min,n_ and *I*_stat,1_ = *I*_min,p_. This results in the inverter efficiency in Eq. (6). *I*_p_/*I*_stat_ for the perfectly matched CMOS is given in Eq. (7) using *I*_max,n_ = *I*_max,p_ = *I*_max_ and *I*_min,n_ = *I*_min,p_ = *I*_min_.

6 $$CMOS{:}\, \frac{{I}_{p}}{{I}_{stat}}=\frac{\frac{{I}_{max,n}{I}_{max,p}}{{I}_{max,n}+{I}_{max,p}}}{{I}_{min,n}+{I}_{min,p}}$$

7 $$perfectly\,\, matched\,\, CMOS{:}\, \frac{{I}_{p}}{{I}_{stat}}=\frac{\frac{{I}_{max}{I}_{max}}{{I}_{max}+{I}_{max}}}{{I}_{min}+{I}_{min}}=\frac{{0.5I}_{max}}{2{I}_{min}}=\frac{{I}_{max}}{4{I}_{min}}\propto \frac{{I}_{max}}{{I}_{min}}$$

For the perfectly matched inverter in Fig. 3d,h,l, *I*_*p*_ = *0.*5*I*_max,n_ = 2.7 µA, *I*_stat_ = 2*I*_min,n_ = 800 pA, *I*_p_/*I*_stat_ = 3300 (at *V*_DD_ = 5 V). The performance of the SnO TFTs is inferior to that of the a-ISO TFTs and therefore *I*_p_/*I*_stat_ should be somewhere between 1 and 3300 for an a-ISO/SnO CMOS inverter.

In Fig. 3b,f,j the resistor has been replaced with the p-type SnO TFT with (*W/L*)_p_ = 100 to form a complementary inverter with (*W/L*)_p_/(*W/L*)_n_ = 5. Increasing (*W/L*)_p_/(*W/L*)_n_ is common practice^19,20,27^ to compensate for the lower mobility of the p-type TFT. In this case, the output currents are matched at *V*_GS_ = 5 V but the mismatch at *V*_GS_ = 0 V,1 V is increased, as shown in Fig. 3b. The VTCs show the NMs nearly double (compared to the unipolar case) and at *V*_DD_ = 5 V a rail-to-rail voltage swing is achieved. *I*_stat,1_ = *I*_min,p_ is reduced to 200 nA and *I*_p_ increases to 3.0 µA. At *V*_DD_ = 5 V, this inverter switches four times as fast and *I*_stat_ is reduced by ~ 3.5, resulting in *I*_p_/*I*_stat_ = 15. Therefore, this inverter is 15 times more ‘efficient’ than the unipolar resistive inverter (note this is an underestimate as a TFT generally discharges the load capacitance quicker than a resistor). At *V*_DD_ = 3 V, *I*_p_ is only 810 nA and *I*_p_/*I*_stat_ = 4. For *V*_DD_ = 1 V, *V*_out_ =  ~ 0.7 V at *V*_in_ = 1 V and no inversion is possible; the gain is < 1 resulting in sub-zero NMs.

In Fig. 3c,g,k the inverter is modelled using the p-type SnO TFT with (*W/L*)_p_ = 20 ((*W/L*)_p_/(*W/L*)_n_ = 1). The output currents are no longer matched at *V*_GS_ = 5 V but the match improves at lower *V*_GS_ (Fig. 3c). Figure 3g shows improved performance at lower voltages resulting in rail-to-rail swing and a NM = 44% when *V*_DD_ = 3 V. At *V*_DD_ = 1 V, the gain is just above unity with a small NM = 4% but *V*_out_ > 0.3 V. Interestingly, the NM at *V*_DD_ = 5 V also improves, despite a larger mismatch at *V*_GS_ = 5 V. Crucially, this shows that matching *I*_min,n_ and *I*_min,p_ at the expense of mismatching *I*_max,n_ and *I*_max,p_ improves the NMs over a wide range of *V*_DD_. Moreover, *I*_stat,1_ = *I*_min,p_ is reduced from 200 to 40 nA. *I*_p_ is reduced to 1.1 µA (*V*_DD_ = 5 V) and 350 nA (*V*_DD_ = 3 V) causing a longer propagation delay. However, the overall efficiency is higher since *I*_p_/*I*_stat_ is ~ 26 and ~ 9 for *V*_DD_ = 5 V and *V*_DD_ = 3 V respectively. This shows that reducing (*W/L*)_p_ from 5 to 1 nearly doubles the efficiency, and improves the NMs. Compared to ~ 3300 for the ideally matched case, 26 is relatively low, but is still > 25-fold improvement over the unipolar resistive inverter. Since the power consumption of recent flexible microprocessors employing resistive load technology is > 99% static^14^, a 25-fold reduction in *P*_stat_ could potentially result in a 25-fold increase in the number of gates. An additional advantage is that (*W/L*)_p_/(*W/L*)_n_ = 1 reduces the area occupied by the CMOS inverter, as well as parasitic capacitance. Whether these advantages outweigh the complexity of adding the p-type (n-type) material varies for different applications. Note that Si processors operate at higher frequencies since the mobility of crystalline Si is > 100 times higher than for metal-oxides and organics. Combined with the low off-current of Si transistors (resulting in a high *I*_p_/*I*_stat_), this means *P*_stat_ of Si CMOS gates is usually negligible compared to the dynamic power (*P*_dyn_) as defined in Eq. (8). Further note that *P*_dyn_ scales with *f* (and therefore *I*_p_) which makes it impossible to optimise *I*_p_/*I*_dyn_ in Eq. (9) (for constant *V*_DD_, *C*_L_ and assuming the input rise time, *t*_s_, is negligible). For this reason the inverter efficiency is defined as *I*_p_/*I*_stat_. The type of application also matters, for example in a microprocessor the average number of gates that switch at any time is a relatively low percentage (*x* in Eq. (9)) of the total number of gates which reduces *P*_*dyn*_. In less complex systems, a higher proportion of gates might be switching and *x* will be larger.

8 $${P}_{tot}={P}_{dyn}+{P}_{stat}={x(C}_{L}{{V}_{DD}}^{2}+{V}_{DD}{I}_{peak}{t}_{s})f+{V}_{DD}\frac{{I}_{stat}}{2}$$

9 $$\frac{{P}_{tot}}{{V}_{DD}}={x(C}_{L}{V}_{DD}+{I}_{peak}{t}_{s})f+\frac{{I}_{stat}}{2}={xI}_{dyn}+\frac{{I}_{stat}}{2}$$

## All-oxide CMOS inverter performance

**Figure 4:**
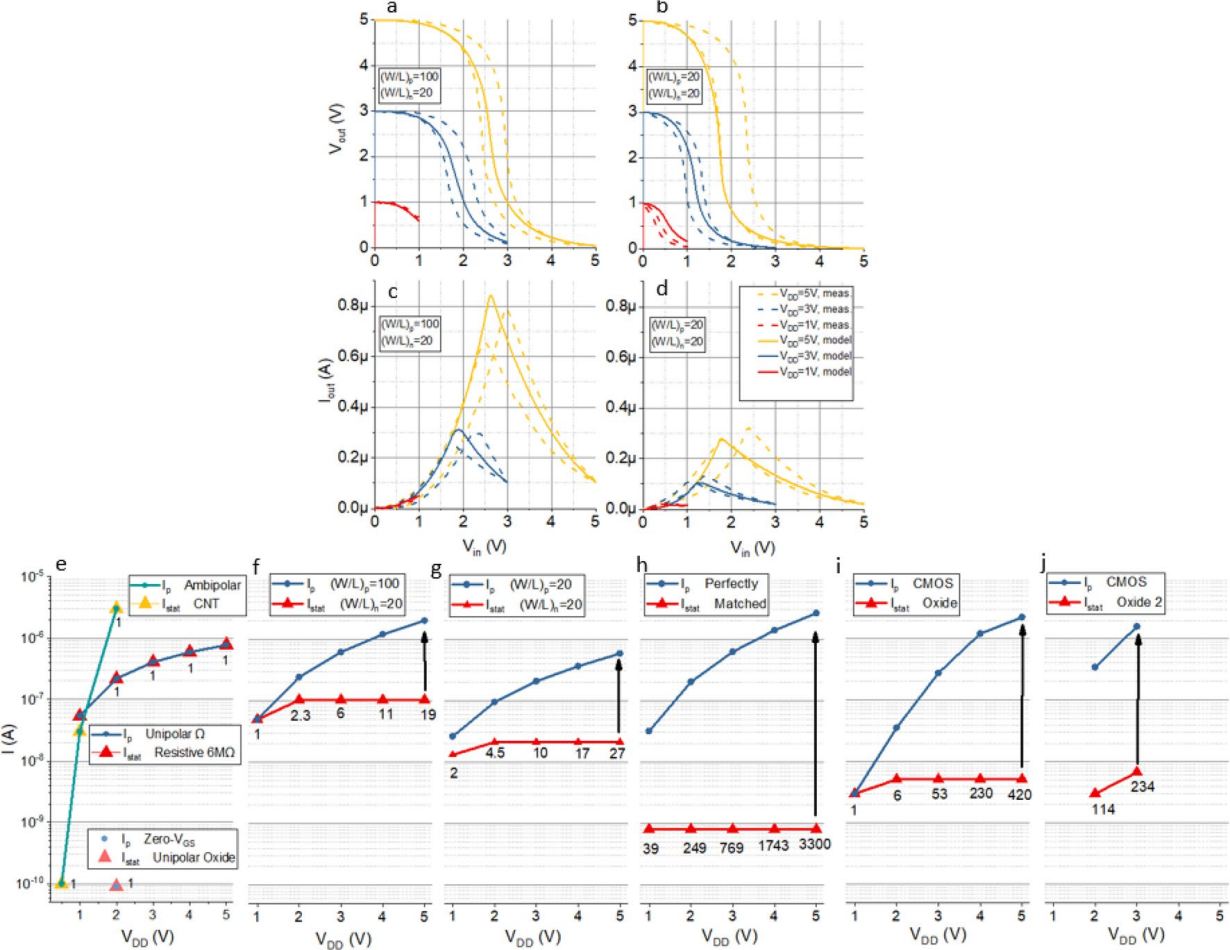
Measured and modelled (solid line) voltage transfer characteristics for the a-ISO + SnO inverters (**a**,**b**) and current transfer characteristics (**c**,**d**). Peak, static currents and the ratio between them for *V*_DD_ = [0,5 V] of different inverter configurations (**e**) unipolar resistive, CNT ambipolar^28^, IGZO zero-*V*_GS_^29^, (**f**) CMOS a-ISO + SnO *(W/L)*_p_*/(W/L)*_n_ = 100/20, (**g**) CMOS a-ISO + SnO *(W/L)*_p_*/(W/L)*_n_ = 20/20, (**h**) CMOS ISO perfectly matched, (**i**) CMOS IGZO + SnO^22^, (**j**) CMOS IGZO + SnO^30^.

The modelling results were verified experimentally by connecting SnO and a-ISO TFTs to form two all-metal-oxide CMOS inverters with different (*W/L*)_p_/(*W/L*)_n_. The first inverter consists of the a-ISO TFT with (*W/L*)_n_ = 20 and the SnO TFT with (*W/L*)_p_ = 100. In the second configuration the SnO TFT with (*W/L*)_p_ = 20 is used. The measured VTCs and CTCs are represented by the dotted lines in Fig. 4a,b,c,d. The difference between the forward and backward sweeps is caused by the hysteresis of the SnO TFT (Fig. 2b,c).

Figure 4a–d show the model fits the measured data well for both configurations across a range of *V*_DD_. It should be noted that a correction was applied to account for a reduction in measured *I*_max,p_ and *I*_min,p_, caused by hysteresis from sweeping *V*_GS_ = *V*_in_. For (*W/L*)_p_ = 20 and *V*_DD_ = 1 V, the measured device achieves rail-to-rail swing and outperforms the model due to the reduced *I*_min,p_. The Supplementary Information provides details on the modelling and hysteresis effects.

Figure 4f–h shows *I*_p_, *I*_stat_ and *I*_p_/*I*_stat_ of the two measured devices and perfectly matched inverter for *V*_DD_ = [0, 5 V]. By changing (*W/L*)_p_ from 100 to 20, *I*_stat_ is reduced by a factor 5 while *I*_p_ is only reduced by 3; *I*_p_/*I*_stat_ therefore improves by 60%. This confirms that reducing (*W/L*)_p_ increases *I*_p_/*I*_stat_ compared to simply scaling up (*W/L*)_p_ inversely with mobility as long as (i) *I*_min,p_ scales with (*W/L*)_p_ and (ii) *I*_min,p_ is considerably larger than *I*_min,n_.

Figure 4h shows *I*_p_/*I*_stat_ for the perfectly matched CMOS inverter reaches ~ 3300 for *V*_*DD*_ = 5 V due to the low *I*_stat_. For CMOS devices, *I*_p_ scales with *V*_DD_ while *I*_stat_ remains approximately constant. Therefore, *I*_p_/*I*_stat_ increases and the inverter becomes more ‘efficient’ at higher *V*_DD_. This key advantage of CMOS over other inverters is quantitively captured by *I*_p_/*I*_stat_.

Figure 4e shows *I*_p_/*I*_stat_ is ~ 1 for the (6 MΩ) unipolar resistive inverter since *I*_stat_ =  ~ *I*_p_ as *V*_DD_ increases. Two other inverter configurations that commonly employ TFTs are shown; an ambipolar inverter^28^ and zero-*V*_GS_ metal-oxide inverter^29^. Both these devices achieve ultralow power consumption (< 1 nW) but their use is limited since *I*_p_/*I*_stat_ = 1. The ambipolar inverter operates down to 0.5 V where *I*_stat_ = 100 pA but since *I*_p_ = *I*_stat_ the operating frequency is ~ 10 Hz, limiting the range of applications. The device can operate at higher voltages but *I*_stat_ increases with *I*_p_, as for the unipolar resistive inverter. In this case, the complementary configuration improves the VTCs and NMs but not *I*_p_/*I*_stat_. The zero-*V*_GS_ inverter combines two Schottky-barrier IGZO TFTs. The pull-up TFT operates in saturation with *V*_GS_ = 0 V resulting in an ultralow operating current of ~ 100 pA for *V*_DD_ = 2 V. This is similar to operating with a large resistive load (*I*_p_/*I*_stat_ =  ~ 1) and the switching frequency is once again limited to ~ 10 Hz. Both the ambipolar and zero-*V*_GS_ configurations are suitable for ultralow-power, low-frequency applications but not for flexible processors where a high *I*_p_/*I*_stat_ is required for operation > 1 kHz and low *P*_stat_.

In Fig. 4i,j the *I*_p_/*I*_stat_ of two state-of-the-art all-oxide CMOS inverters have been calculated and plotted for comparison^22,30^. The SnO TFTs reported in these works have an *I*_on_/*I*_off_ of ~ 10^5^ and *SS* of ~ 1 V/dec, which are superior to our SnO TFTs (likely due to thin high-quality Al_2_O_3_ dielectric layers). As expected, *I*_p_/*I*_stat_ of these inverters is considerably higher but for both devices (*W/L*)_p_/(*W/L*)_n_ has been scaled up using the silicon CMOS approach (to 5 and 3 respectively) to simply compensate for the mobility difference. In the next section, we explain how *I*_p_/*I*_stat_ for these inverters can be improved by scaling (*W/L*)_p_/(*W/L*)_n_ optimally.

## Optimal geometric aspect ratio

In this work we have shown that reducing (*W/L*)_p_/(*W/L*)_n_ can increase *I*_p_/*I*_stat_ compared to simply scaling up *W/L* inversely with mobility, while reducing the area. This raises the question whether an optimal (*W/L*)_p_/(*W/L*)_n_ exists, which maximises these parameters. Ideally the maximum (*V*_GS_ =  ± *V*_DD_) and minimum (*V*_GS_ = 0 V) drain currents of the n-type TFTs, *I*_max,n_ and *I*_min,n_, equal the ones of the p-type TFTs, *I*_max,p_ and *I*_min,p_. For silicon transistors with full channel depletion *I*_min,p_ and *I*_min,n_ are usually similar and independent of (*W/L*). This means the lower hole mobility is compensated by scaling (*W/L*)_p_ up until ((*W/L*)_p_*I*_max,p_) matches *I*_max,n_ (Fig. 1b,g). As long as *V*_th,p_ and *V*_th,n_ are well matched, and the oxide capacitance is uniform, an excellent match of the output characteristics is achieved. As explained previously, this does not work for metal-oxide TFTs since the p-type (SnO) TFT has a high *I*_min,p_ and low *I*_max,p_ compared to the n-type (a-ISO) TFT as illustrated in Fig. 1i. Scaling up (*W/L*)_p_ brings (*W/L*)_p_*I*_max,p_ closer to *I*_max,n_ but increases the gap between (*W/L*)_p_*I*_min,p_ and *I*_min,n_. Here we propose the optimal (*W/L*)_p_ can be found by setting the ratios *I*_max,n_/(*W/L*)_p_*I*_max,p_ and (*W/L*)_p_*I*_min,p_/*I*_min,n_ equal (assuming *I*_max,p_ and *I*_min,p_ correspond to (*W/L*)_p_ = 1). As such, a compromise is made between matching minimum and maximum output currents. The optimal (*W/L*)_p_ can be found by solving:

10 $$\frac{{I}_{\mathrm{max},\mathrm{n}}}{ {\left(\frac{W}{L}\right)}_{\mathrm{p}}{I}_{\mathrm{max},\mathrm{p}}} = \frac{{\left(\frac{W}{L}\right)}_{\mathrm{p}}{I}_{\mathrm{min},\mathrm{p}}}{{I}_{\mathrm{min},\mathrm{n}}}$$

This value is dependent on *V*_*DD*_ since the output characteristics vary with *V*_DS_. For silicon transistors *I*_min,p_ no longer scales with (*W/L*)_p_ and equals ~ *I*_min,n_; therefore (*W/L*)_p_*I*_min,p_/*I*_min,n_ can be set to ~ 1. This results in an optimal (*W/L*)_p_ equal to *I*_max,n_/*I*_max,p_ = *μ*_sat,n_/*μ*_sat,p_ as expected:

11 $$Silicon{:}\,\, optimal {\left(\frac{W}{L}\right)}_{\mathrm{p}}=\frac{{I}_{\mathrm{max},\mathrm{n}}}{{I}_{\mathrm{max},\mathrm{p}}} = \frac{{\mu }_{\mathrm{sat},\mathrm{n}}}{{\mu }_{\mathrm{sat},\mathrm{p}}}$$

For TFTs where *I*_min,p_ (or *I*_min,n_ for organics) scales with (*W/L*)_p_ ((*W/L*)_n_), corresponding to the scenarios in Fig. 1c,d,e,h, the following are applicable:

12 $$Oxide{:}\,\, optimal {\left(\frac{W}{L}\right)}_{\mathrm{p}}=\sqrt{\frac{{I}_{\mathrm{max},\mathrm{n}}}{{I}_{\mathrm{max},\mathrm{p}}}\times \frac{{I}_{\mathrm{min},\mathrm{n}}}{{I}_{\mathrm{min},\mathrm{p}}}}$$

13 $$Organics{:}\,\, optimal {\left(\frac{W}{L}\right)}_{\mathrm{n}}=\sqrt{\frac{{I}_{\mathrm{max},\mathrm{p}}}{{I}_{\mathrm{max},\mathrm{n}}}\times \frac{{I}_{\mathrm{min},\mathrm{p}}}{{I}_{\mathrm{min},\mathrm{n}}}}$$

When calculating the optimal (*W/L*), it should be noted that *I*_min,p_ and *I*_max,p_ in Eq. (12) are the normalised values corresponding to (*W/L*)_p_ = 1 (similarly *I*_min,n_ and *I*_max,n_ in Eq. (13) correspond to (*W/L*)_n_ = 1). By taking the derivative of *I*_p_/*I*_stat_ w.r.t. (*W/L*)_p_ and setting this equal to zero, we show that the expression in Eq. (12) indeed maximises *I*_p_/*I*_stat_ (for (*W/L*)_p_ > 0). The expression for *I*_p_/*I*_stat_ for a CMOS inverter where both *I*_min,p_ and *I*_max,p_ scale with (*W/L*)_p_ is given in Eq. (14). Equation (15) shows that the optimal (*W/L*)_p_ from Eq. (12) sets the top part of the derivative of *I*_p_/*I*_stat_ with respect to (*W/L*)_p_ equal to zero. In the Supplementary Information a plot of *I*_p_/*I*_stat_ versus (*W/L*)_p_ is included for the inverters in this work (Fig. S4).

14 $$\frac{{I}_{p}}{{I}_{stat}} =\frac{\frac{{I}_{max,n}{\left(\frac{W}{L}\right)}_{\mathrm{p}}{I}_{max,p}}{{I}_{max,n}+{\left(\frac{W}{L}\right)}_{\mathrm{p}}{I}_{max,p}}}{{I}_{min,n}+{\left(\frac{W}{L}\right)}_{\mathrm{p}}{I}_{min,p}}$$

15 $$\frac{\partial }{\partial {\left(\frac{W}{L}\right)}_{\mathrm{p}}}\frac{{I}_{p}}{{I}_{stat}} =\frac{{I}_{max,n}{I}_{max,p}({I}_{max,n}{I}_{min,n}-{I}_{max,p}{I}_{min,p}{{\left(\frac{W}{L}\right)}_{\mathrm{p}}}^{2})}{{\left({I}_{max,p}{\left(\frac{W}{L}\right)}_{\mathrm{p}}+{I}_{max,n}\right)}^{2}+ {\left({I}_{min,n}+{I}_{min,p}{\left(\frac{W}{L}\right)}_{\mathrm{p}}\right)}^{2}}$$

It is suggested to calculate the optimal (*W/L*)_p_ ((*W/L*)_n_) for the maximum required *V*_*DD*_. If *I*_min,n_ (*I*_min,p_) is much smaller than other currents in the system, (i.e. for IGZO TFTs *I*_min,n_ can be ~ 1fA) it is advised to set *I*_min,n_ (*I*_min,p_) equal to the lowest critical current. This method can also be applied to maximise *I*_p_/*I*_stat_ for Si CMOS inverters. However, the low *I*_stat_ means the effect on the overall power consumption will be negligible and it makes more sense to scale (*W/L*)_p_ to maximise NMs.

**Figure 5:**
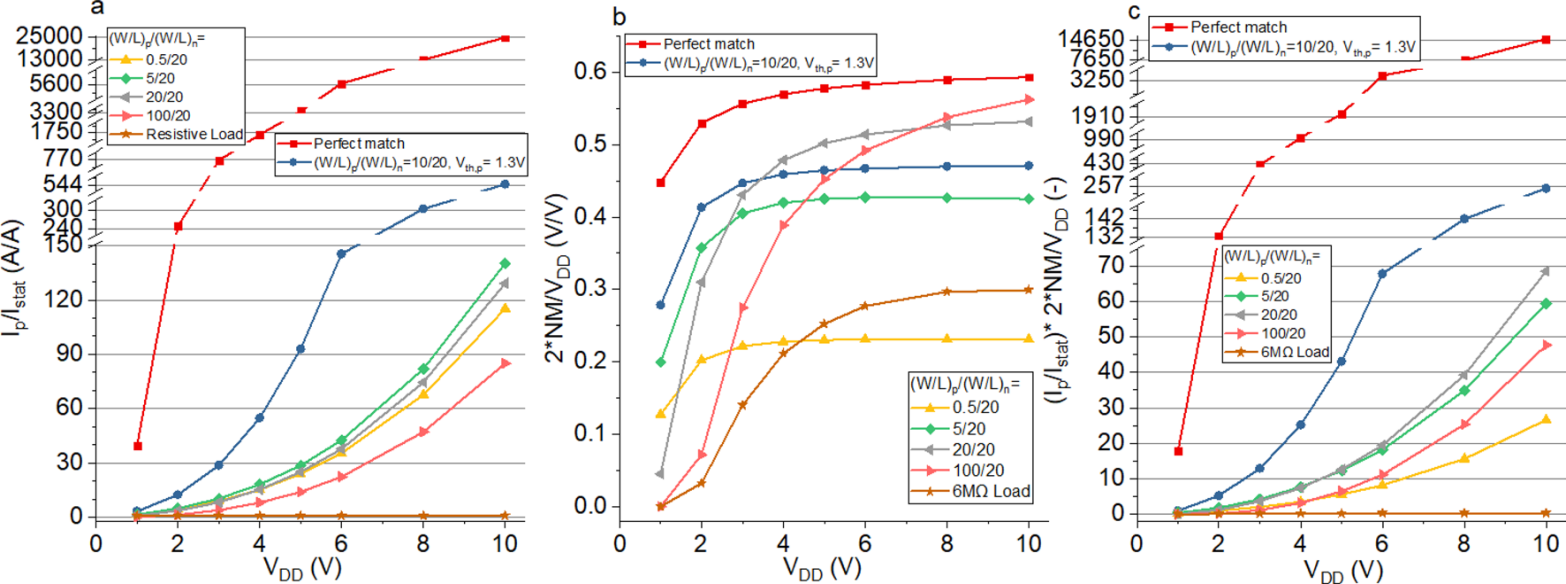
(**a**) *I*_p_/*I*_stat_ and (**b**) normalised noise margin (2*NM/*V*_DD_) and (**c**) (*I*_p_/*I*_stat_)*(2*NM/*V*_DD_) for the unipolar resistive load inverter, CMOS ISO-SnO inverter with different (*W/L*) ratios, threshold voltage shifted and perfectly matched cases.

For the a-ISO-SnO inverter with (*W/L*)_n_ = 20, the optimal (*W/L*)_p_ were calculated as 4 and 5 at *V*_DD_ = 5 V and *V*_DD_ = 10 V respectively. Using the model, *I*_p_/*I*_stat_ and the NMs were estimated for different (*W/L*)_p_. Figure 5a shows reducing (*W/L*)_p_ from 100 to 5, nearly doubles *I*_p_/*I*_stat_ for *V*_DD_ = 5 V and improves it by over 60% for *V*_DD_ = 10 V. However, reducing (*W/L*)_p_ any further from 5 to 0.5 leads to a decrease in *I*_p_/*I*_stat_ and 5 is therefore the optimal value. Increasing (*W/L*)_p_ from 5 to 20 reduces *I*_p_/*I*_stat_ by only 10%. The perfectly matched and resistive load cases are shown for comparison. It also includes the scenario where *V*_th,p_ is reduced by 1 V to 1.3 V resulting in a considerable reduction in *I*_min,p_ and therefore increase in *I*_p_/*I*_stat_. This shows changing (*W/L*)_p_ makes a noticeable difference but combining it with an optimal *V*_th,p_ can have significantly greater impact (note the optimal (*W/L*)_p_ is 10 in this case). The *V*_th_ of oxide TFTs is difficult to control as there is no appropriate doping process. It can be improved by reducing the dielectric thickness (and using a high k dielectric), and removing defects between the dielectric and semiconductor interface (i.e. by annealing, interface layers, choice of materials and processing conditions).

NMs are equally important and modelled as a function of (*W/L*)_p_ (Fig. 5b) which confirms that reducing (*W/L*)_p_ from 100 to 20 increase the NMs for *V*_DD_ < 8 V. For (*W/L*)_p_ = 5 the NMs improve for *V*_DD_ < 3 V but a further reduction to (*W/L*)_p_ = 0.5 lowers them for all *V*_DD_. NMs for the perfectly matched and resistive load are included for reference.

To take into account both *I*_p_/*I*_stat_ and the NMs, a new dimensionless parameter is introduced, (*I*_p_/*I*_stat_)*NM/(*V*_DD_/2), and plotted in Fig. 5c. This shows that for *V*_DD_ < 5 V the performance of (*W/L*)_p_ = 5 and (*W/L*)_p_ = 20 is very close (and nearly double that of (*W/L*)_p_ = 0.5 and (*W/L*)_p_ = 100). For *V*_DD_ > 5 V, (*W/L*)_p_ = 20 outperforms (*W/L*)_p_ = 5 as the NM increases more than *I*_p_/*I*_stat_. This shows that (*W/L*)_p_ = 5 represents a lower bound which maximises *I*_p_/*I*_stat_ but that it might be worth increasing (*W/L*)_p_ to above this value to increase NMs further. Alternatively a larger (*W/L*)_p_ might be required to achieve higher switching frequency. In this case we suggest to increase (*W/L*)_n_ first (and therefore *I*_max,n_) and find the corresponding optimal (*W/L*)_p_. For example, the inverter with (*W/L*)_p_/(*W/L*)_n_ = 60/60 achieves the same average switching current (*I*_p_) as (*W/L*)_p_/(*W/L*)_n_ = 100/20 while the sum of the static currents is only half (improving *I*_p_/*I*_stat_ by a factor two with the same total area at *V*_DD_ = 5 V).

Table 1 shows the actual and suggested optimal (*W/L*)_p_/(*W/L*)_n_, corresponding output currents, and *I*_p_/*I*_stat_. For the inverter in Fig. 4i^22^, *I*_p_/*I*_stat_ can almost be tripled by choosing the optimal (*W/L*)_p_/(*W/L*)_n_, while reducing the total area. This represents a straightforward method to geometrically optimise the inverter performance, provided the maximum and minimum output currents of the n- and p-type TFTs are measured (or modelled). Calculating NMs is more difficult but can be obtained by adjusting the modelling parameters in Eqs. (1) and (2). *I*_p_/*I*_stat_ for the inverter in Fig. 4j^30^ is close to the optimal value and therefore the gains are limited. Optimisation based on NMs and switching speed is likely to be more important in this case. For the SnO-ZnO CMOS inverter^27^ *I*_p_/*I*_stat_ is only 1.8. This inverter has excellent NMs (> 60%), but *I*_p_/*I*_stat_ shows that the inverter efficiency is only marginally better than the unipolar resistive inverter. The last row contains an estimate for the optimal (*W/L*)_p_/(*W/L*)_n_ for an organic complementary inverter^11^ for which the p-type TFT outperforms the n-type. However, note that for this inverter *I*_p_/*I*_stat_ is already > 1000, meaning it is unlikely that *P*_stat_ is the dominant factor.

**Table 1:** Suggested optimal geometric aspect ratios.

Channel		*V*_DD_ (V)	Total area	(*W/L*)_p_ (*W/L*)_n_	$\frac{{I_{{{\text{min}},{\text{p}}}} \;{\text{(A)}}}}{{I_{{{\text{min}},{\text{n}}}} \;{\text{(A}})}}$	$\frac{{I_{{{\text{max}},{\text{n}}}} \;{\text{(A)}}}}{{I_{{{\text{max}},{\text{p}}}} \;{\text{(A}})}}$	$\frac{I_{{\text{p}} \;{\text{(A)}}}}{{I_{{{\text{stat}}}} \;{\text{(A}})}}$	References	Total area	(*W/L*)_p_ (*W/L*)_n_	$\frac{{{I}_{{{\text{min}},{\text{p}}}} \;{\text{(A)}}}}{{I_{{{\text{min}},{\text{n}}}} \;{\text{(A}})}}$	$\frac{{I}_{{{\text{max}},{\text{n}}} \;{\text{(A)}}}}{{I_{{{\text{max}},{\text{p}}}} \;{\text{(A}})}}$	$\frac{{I}_{{\text{p}}} \;{\text{(A)}}}{{I_{{{\text{stat}}}} \;{\text{(A}})}}$
p	n		(*W/L*)_p_+(*W/L*)_n_						(*W/L*)_p_+(*W/L*)_n_				
Actual values									Suggested optimal values (*perfectly matched*)				
SnO	a-ISO	10	120	100	$\frac{{{2}00{\text{n}}}}{{0.4{\text{n}}}} = 500$	$\frac{40\upmu }{{31\upmu }} = 1.3$	$\frac{17\upmu }{{0.2\upmu }} = 85(110)*$	This work	25 (*40*)	5 (*20*)	$\frac{{10{\text{n}}}}{{0.4{\text{n}}}} = 25$	$\frac{40\upmu }{{1.6\upmu }} = 25$	$\frac{1.5\upmu }{{10{\text{n}}}} = 148({\it 25{,}000})$
				20						20			
		5	40	20	$\frac{{40{\text{n}}}}{{0.4{\text{n}}}} = 100$	$\frac{5.4\upmu }{{1.3\upmu }} = 4$	$\frac{1.1\upmu }{{40{\text{n}}}} = 26(28)*$		24 (*40*)	4 (*20*)	$\frac{{8{\text{n}}}}{{0.4{\text{n}}}} = 20$	$\frac{5.4\upmu }{{0.26\upmu }} = 21$	$\frac{0.25\upmu }{{8.4{\text{n}}}} = 30({\it 3300})$
				20						20			
SnO	ZnO	8	6	5	$\frac{{750{\text{n}}}}{{0.3{\text{n}}}} = 2500$	$\frac{2.6\upmu }{{2.7\upmu }} = 1$	$\frac{1.3\upmu }{{0.8\upmu }} = 1.7$	^27^	1.1 (*2*)	0.1 (*1*)	$\frac{{15{\text{n}}}}{{0.3{\text{n}}}} = 50$	$\frac{2.6\upmu }{{0.05\upmu }} = 50$	$\frac{0.05\upmu }{{15{\text{n}}}} = 3.5({\it 2200})$
				1						1			
SnO	IGZO	5	7	6	$\frac{{5{\text{n}}}}{{0.1{\text{n}}}} = 50$	$\frac{3\upmu }{{8\upmu }} = 0.4$	$\frac{2.2\upmu }{{5.1{\text{n}}}} = 420$	^22^	1.5 (*2*)	0.5 (*1*)	$\frac{{0.4{\text{n}}}}{{0.1{\text{n}}}} = 4$	$\frac{3\upmu }{{0.7\upmu }} = 4$	$\frac{0.6\upmu }{{0.5{\text{n}}}} = 1100({\it 7500})$
				1						1			
SnO	IGZO	2	4	3	$\frac{{3{\text{n}}}}{{0.1{\text{n}}}} = 30$	$\frac{0.8\upmu }{{0.6\upmu }} = 1.3$	$\frac{0.4\upmu }{{3.1{\text{n}}}} = 114$	^30^	1.66 (*2*)	0.66 (*1*)	$\frac{{0.66{\text{n}}}}{{0.1{\text{n}}}} = 6.6$	$\frac{0.8\upmu }{{0.12\upmu }} = 6.6$	$\frac{0.1\upmu }{{0.76{\text{n}}}} = 137({\it 2000})$
				1						1			
Pentacene organic	F_16_CuPc organic	1.5	36.6	3.3	$\frac{{1{\text{p}}}}{{0.1{\text{n}}}} = 0.01$	$\frac{0.6\upmu }{{0.3\upmu }} = 2$	$\frac{0.2\upmu }{{0.1{\text{n}}}} = 1980$	^11^	5.7 (*6.6*)	3.3	$\frac{{1{\text{p}}}}{{7{\text{p}}}} = 0.14$	$\frac{{42{\text{n}}}}{0.3\upmu } = 0.14$	$\frac{0.04\upmu }{{8{\text{p}}}} = 4600({\it 75{,}000})$
				33.3						2.4 (*3.3*)			

*Hysteresis.

## Conclusions (and outlook for all-oxide CMOS inverters)

We have proposed an optimal (*W/L*)_p_/(*W/L*)_n_ that maximises *I*_p_/*I*_stat_ and can be applied universally to silicon, metal-oxide and organic complementary inverters. Notably, our results show that reducing *W/L* of metal-oxide p-type TFTs increases *I*_p_/*I*_stat_ while reducing the area compared to simply scaling up *W/L* inversely with mobility. A high inverter efficiency is critical to reduce *P*_stat_ and increase the gate density of (flexible) processors; we have shown that *I*_p_/*I*_stat_ of state-of-the-art all-oxide CMOS inverters can be maximised by adopting the optimal (*W/L*)_p_/(*W/L*)_n_. In this way n- and p-type TFTs with significant differences in performance can be matched optimally without changing the intrinsic properties such as *V*_th_, SS, *I*_on_/*I*_off_ and mobility.

Despite the clear need for all-oxide CMOS inverters required for low-cost flexible electronics, only a handful have been reported. For ambipolar, unipolar resistive and zero-V_GS_ configurations *I*_p_/*I*_stat_ = 1; they simply cannot provide the necessary reduction in *I*_stat_ without sacrificing switching speed. The high *I*_off_ of SnO TFTs remains a bottleneck, but has reached a sufficiently low level to reduce *I*_stat_ by at least 100-fold over unipolar logic for *V*_DD_ ~ 3–5 V. While this might seem small compared to a ratio of ~ 3300 for a perfectly matched CMOS inverter, it makes a significant difference for natively flexible microprocessors. The recently reported microprocessor containing 18,334 NAND2 gates with unipolar n-type resistive load logic operates at a maximum clock-frequency of 29 kHz^14^. The power consumption is ~ 21 mW, of which > 99% is static. Based on these figures and *V*_DD_ = 3 V, we estimate *I*_p_ = *I*_stat,1_ for each gate to be ~ 750 nA which is close to *I*_p_ values reported in this work and recent literature. This shows that replacing the resistive load with a state-of-the-art SnO TFT, combined with the correct *W/L* ratio, could reduce *I*_stat_ by a factor > 200 at *V*_DD_ = 3 V (constant *I*_p_). Since *P*_stat_ is the dominant factor, this reduces total power by nearly 200-fold, potentially increasing the number of gates on the chip to over a million, approaching VLSI standards. This shows that while the performance of current SnO TFTs is still significantly behind their n-type counterpart, they are already able to reduce *P*_stat_ to such a level that it is no longer the dominant component. A further reduction of *I*_off_ is welcome but will not affect the overall power consumption significantly as the dynamic power will start to dominate. To realise a flexible microprocessor employing all-oxide CMOS technology it is critical to improve the stability (hysteresis) and repeatability of SnO TFTs.

## Methods

### Device fabrication

The CMOS inverters are formed by interconnecting a bottom-gate staggered n-type a-ISO TFT to a bottom-gate staggered p-type SnO TFT. The schematic structure of each TFT is shown in Fig. 2d,i. The substrate of the n-type TFT is glass on which a 100 nm Cr bottom electrode layer is deposited using a Metallifier Sputter Coating System (Precision Atomics). The gate electrode was patterned using Cr etchant. On top of this a 180 nm Al_2_O_3_ dielectric layer (47 nF/cm^2^) was deposited at 150 °C by atomic layer deposition (MVSystems). After this the 10 nm ISO (10 wt% Si) channel layer was deposited by rf-sputtering (MVSystems) at an oxygen-to-argon flow ratio of 16.7%, rf power of 150 W, and deposition pressure of 4 mTorr. The top layer is a 100 nm Mo deposited by the Metallifier Sputter Coating System to form source/drain (S/D) electrodes. Patterning of this layer was done by reactive ion etching (Philips RIE) at a power of 100 W and pressure of 150 mTorr. All layers were patterned with AZ5214E photoresist. The a-ISO devices were annealed for two hours in ambient air at 200 °C to improve the performance. The *W/L* ratio of the devices was 20 with a constant channel length of 20 μm.

For the p-type TFTs, 10 nm SnO layers were formed on thermally-grown SiO_2_ on a p^+^-Si substrate by ALD (Beneq TFS-200) at 170 °C using a tin(II) amide precursor. The p^+^-Si and SiO_2_ (∼ 200 nm) were used as a common gate electrode and a gate insulator (12 nF/cm^2^), respectively. A 100 nm Au layer was deposited as S/D electrodes using a thermal evaporator (Edwards E306A). Finally, a 70 nm passivation layer of Al_2_O_3_ was deposited. The devices were annealed for two hours at 200 °C in ambient air. The active layers and S/D electrodes were patterned by a lift-off process using AZ5214E photoresist. The *W/L* ratio varied from 20 to 100 with a constant channel width of 1000 μm.

The maximum fabrication temperature of the above processes is 200 °C resulting in low fabrication costs and the ability to deposit on flexible substrates such as DuPont Kapton HN sheet. A further reduction in annealing temperature to 170 °C should be possible by increasing the annealing time. Note a p^+^-Si substrate with SiO_2_ was used for the p-type device for ease of manufacturing but in the future this could easily be replaced by a glass or flexible substrate with oxide dielectric.

### Device characterisation

The electrical performance of the TFTs and the inverters were analysed using a Keithley 4200 semiconductor characterisation system. A six probe configuration was used to interconnect the gate electrodes of different n- and p-type TFTs and feed in a common input voltage. Similarly the drain electrodes were interconnected to measure the output voltage. This setup allows to connect p-type TFTs with different *W/L* ratios to the same n-type TFT and isolate the effects of changing (*W/L*)_p_/(*W/L*)_n_.

## Supplementary Information

Supplementary Information.

## Data Availability

The datasets generated during and/or analysed during the current study are available in the Cambridge University Data Repository (http://www.repository.cam.ac.uk/).
