# Supplementary material for: Optimisation of geometric aspect ratio of thin film transistors for low-cost flexible CMOS inverters and its practical implementation
Source: Sci Rep. 2022 Sep 27;12:16111. doi: 10.1038/s41598-022-19989-6 (PMC9515102; doi:10.1038/s41598-022-19989-6)
Supplement: Supplementary file 1 — Supplementary Information. [file 41598_2022_19989_MOESM1_ESM.docx]

**Supplementary Information**

**Illustration of static states and dynamic transitions of CMOS inverter**

In Fig. S1 the static states of the CMOS inverter and dynamic low-to-high and high-to-low transitions are illustrated, along with the terminology for the different performance variables used in this work.

Fig. S1 Schematic of load lines of n- and p-type transistors, voltage transfer characteristics and current transfer characteristics of CMOS inverter. Static operating points at *V*_in_=0 V & *V*_out_=*V*_DD_ (top left) and *V*_in_=*V*_DD_ & *V*_out_ =0 V (bottom right). Schematic of dynamic high-to-low transition (bottom left) and low-to-high transition (top right).


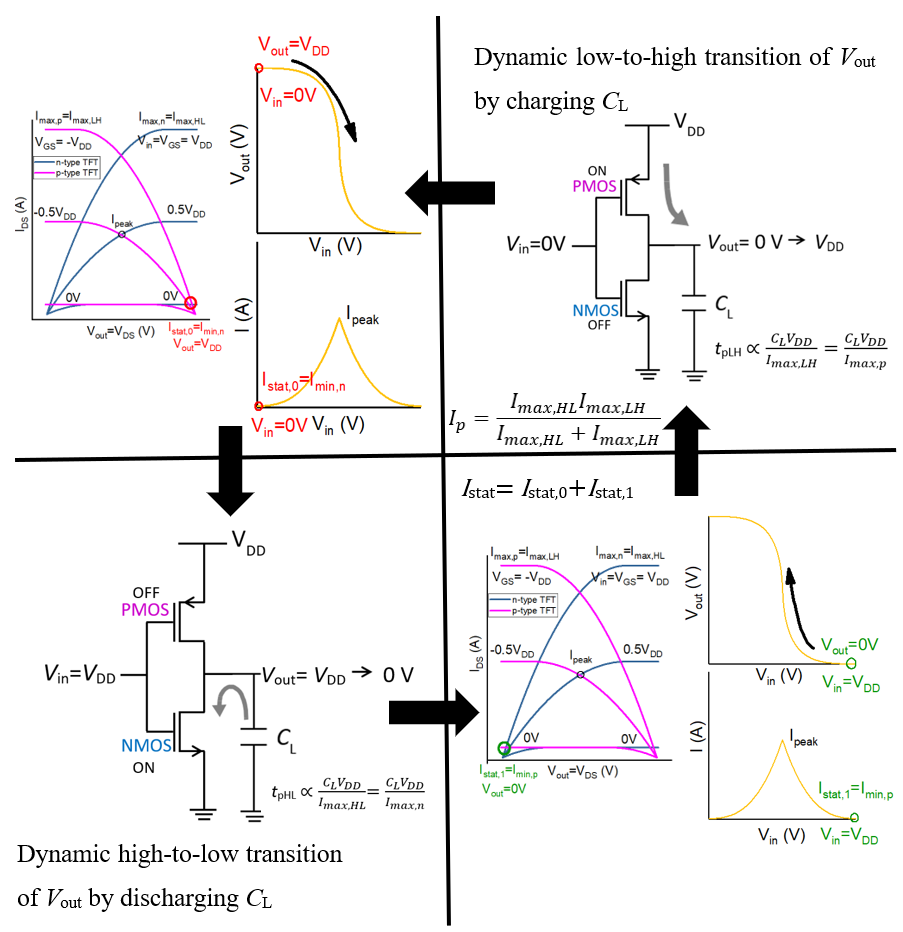


**Modelling**

A model for the output characteristics was developed in MATLAB based on the standard MOSFET equations modified by a prefactor, $\alpha\left( V_{GS}-V_{th,n} \right)$, where *V*_th,n_ is the threshold of the n-type TFT. For standard Si MOSFETs the standard equations fit well as the (saturation) mobility can be assumed to be constant at low gate voltages. Fig. 2a,b shows that for both the a-ISO and SnO TFTs the mobility increases approximately linearly as a function of *V*_GS_ (for *V*_GS_ > *V*_th_) and only starts to saturate at higher gate voltages (*V*_GS_ < -10 V for the SnO TFT, the a-ISO TFT mobility does not start to saturate for *V*_GS_ < 20 V). To create a more accurate fit for the linearly varying mobility in the metal-oxide TFTs, we replaced the constant (saturation) mobility in the standard MOSFET equations by $\alpha\left( V_{GS}-V_{th,n} \right)$. The equations for the a-ISO and SnO TFTs in the linear regime respectively are given in equation the equations below. These equations should provide a better fit for **|***V*_GS_**|** < 10 V (which is the region of interest for CMOS inverters for flexible processors).

$$I_{DS,n}=\left( \frac{W}{L} \right)_{n}C_{ox,n}\alpha\left( V_{\mathrm{GS}}-V_{th,n} \right)\left( \left( V_{\mathrm{GS}}-V_{th,n} \right)V_{\mathrm{DS}}-\beta{V_{\mathrm{DS}}}^{2} \right)+I_{OFF,n} (S1)$$

$$I_{DS,p}=\left( \frac{W}{L} \right)_{p}{(C}_{ox,p}\gamma\left( V_{\mathrm{GS}}-V_{th,p} \right)\left( \left( V_{GS}-V_{th,p} \right)V_{\mathrm{DS}}-\delta{V_{\mathrm{DS}}}^{2} \right)+I_{OFF,p}) (S2)$$

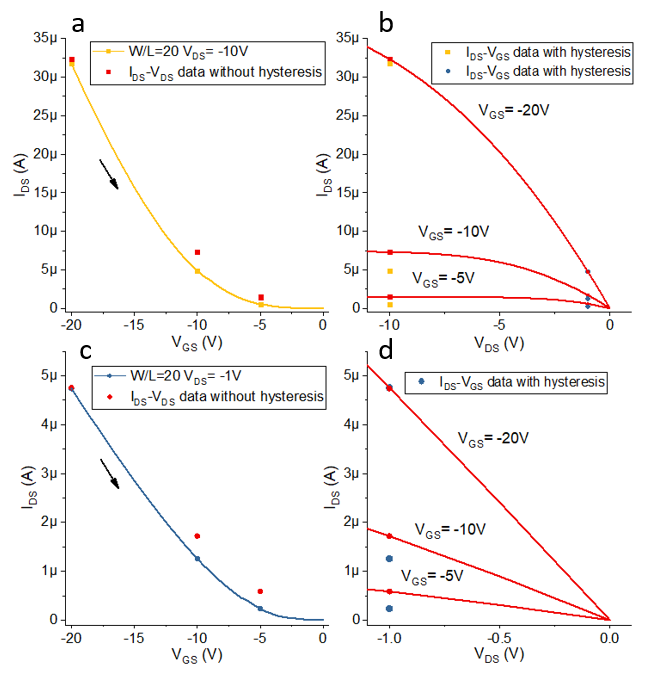
The value for each of the parameters in these equations are given below. For each of the TFTs (*W/L*) is known, *C*_ox_ was measured, *V*_th_ and *I*_OFF_ were extracted from the transfer characteristics in the previous section. The values of $\alpha, \beta, \gamma, \delta$ are obtained using an optimum fit (in OriginPro) based on the measured output characteristics. The parameters for the a-ISO TFT are:

$$\left( \frac{W}{L} \right)_{n}=20, C_{ox,n}=\frac{52 nF}{{cm}^{2}}, \alpha=\frac{0.073 {cm}^{2}}{V^{2}s}, V_{th,n}=-0.2 V, \beta=0.5, I_{OFF,n}=300 pA$$

The parameters for the SnO TFTs are:

$$\left( \frac{W}{L} \right)_{p}=20 \left( 100 \right), C_{ox,p}=\frac{12 nF}{{cm}^{2}}, \gamma=\frac{0.043 {cm}^{2}}{V^{2}s}, V_{th,p}=2.3 V, \delta=0.78, I_{OFF,p}=14 pA$$

In the saturation regime *I*_DS,n_ and *I*_DS,p_ equal the maximum value when $\frac{\partial I_{\mathrm{DS}}}{\partial V_{\mathrm{DS}}}=0$. Fig. 2e,f,g,h show the model closely fits the measured data. The logarithmic graphs in Fig. 2e,f show a good match for lower *V*_GS_ and especially for *I*_min_, which represents the static off-current of the CMOS inverter when *V*_in_ = *V*_GS_ is low (0 V) and high (*V*_DD_) for the n- and p-type devices, respectively. Fig. 2g,h demonstrate a good fit on a linear scale at higher *V*_GS_. The resolution of *V*_DS_ was set to 0.05V for the curves in Fig. 2e,f,g,h. The inverter model simulates the VTC and CTC based on the intersection points of the output characteristics.The resolution of *V*_DS_ and *V*_GS_ were set to 0.05V for the curves in Fig. 3.

Fig. S2. (a, c) SnO transfer characteristics with hysteresis from sweeping V_GS_ from -20 to 0V (yellow, blue). (b, d) Output characteristics without hysteresis (red).

**Hysteresis**

Hysteresis is commonly observed in SnO TFTs due to the high trap state density near the interface between the SnO layer and the gate insulator. In Fig. 2b,c it can be seen that the hysteresis between the forward and backward sweep is over 5V when sweeping between -30 and +30V. Due to the hysteresis there is a difference between the operating points on the *I*_DS_-*V*_DS_ and *I*_DS_-*V*_GS_ curves (Fig. S2). In Fig. S2a,c the yellow and blue curves indicate the operating points of the TFT when the gate is swept from -20V to 0V while *V*_DS_ is kept constant at -10 and -1V respectively. The red squares and circles in Fig. S2a,c show the operating points at *V*_GS_ = [-5, -10, -20V] for the same *V*_DS_ according to the output characteristics which are shown in Fig. S2b,d. For a device without hysteresis the red squares and circles should lie on the yellow and blue curves, i.e. the operating points on the transfer and output curves match each other. In this case the hysteresis effect of sweeping the gate voltage from -20V to 0V is clearly visible; at the starting point *V*_GS_ = -20V the red squares and circles match up with the yellow squares and blue circles, but as soon as *V*_GS_ is swept in the positive direction it becomes clear that *I*_DS_ drops more than we would expect based on the output characteristics. At *V*_GS_ = -10V, *I*_DS_ is 30-40% lower and at *V*_GS_ = -5V the difference is nearly 60%. A similar trend can be observed at *V*_DS_=-1V. In the CMOS inverter the SnO TFT undergoes a similar transition. For example for *V*_DD_ = 20V, *V*_GS_ is -20V when *V*_in_=0V and gradually drops towards 0V as V_in_ reaches *V*_DD_. Simultaneously, *V*_DS_ (which equals *V*_out_) drops from *V*_DD_ to 0V (for rail-to-rail swing) but the hysteresis due to the change in *V*_DS_ is negligible as can be seen in Fig. 2e,f,g,h; the forward and backward sweeps of the output characteristics overlap when *V*_GS_ is kept constant.


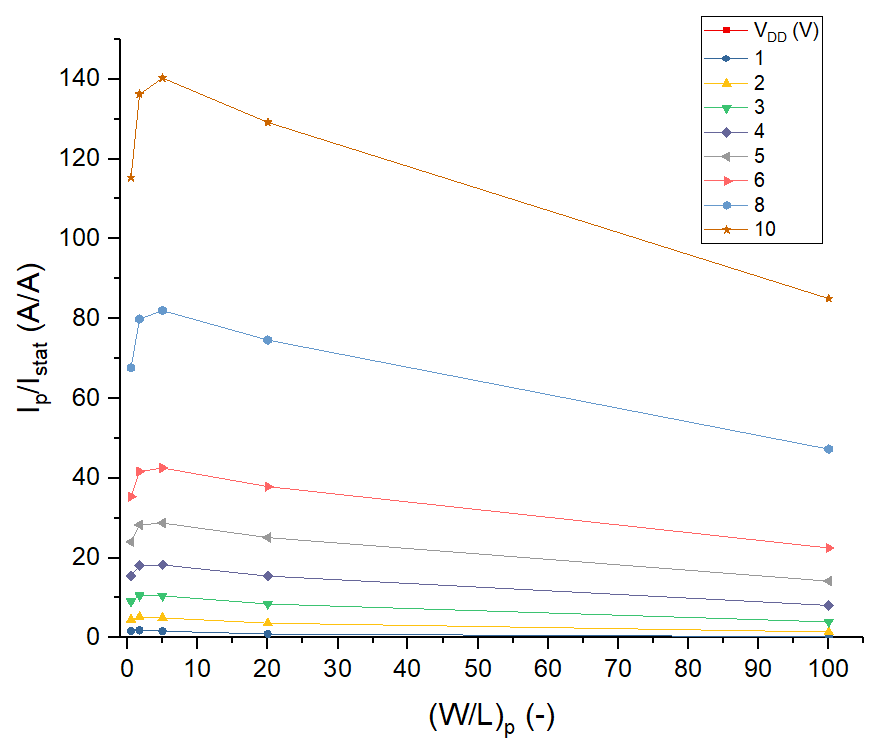
The hysteresis effect (from sweeping *V*_GS_) can be taken into account in the output characteristics model by adjusting the $\gamma$ parameter. Fig. S2b,d show the original output curves in red and the yellow and blue operating points with hysteresis. Based on this it is possible to estimate the actual SnO output curves with hysteresis to be ~50% of the original ones. Therefore, halving $\gamma$ from 0.043 to 0.0215 cm^2^/V^2^s in equation S2, represents a reasonably accurate correction for the hysteresis. For the a-ISO TFTs the hysteresis due to sweeping *V*_GS_ and *V*_DS_ are both minimal and no correction to the a-ISO output curves is required.

The solid curves in Fig. 4a,b,c,d represent the inverter modelling results after the SnO hysteresis correction. The VTCs and thus the NMs improve in both cases compared to Fig. 3 since the reduction in SnO current improves the match at lower *V*_GS_. *I*_stat_ reduces by 50% from 200 nA to 100 nA and 40 nA to 20 nA for geometric aspect ratios of 5 and 1 respectively. Note that *I*_p_ reduces by less than 50% (resulting in an increase in *I*_p_/ *I*_stat_). In this case *I*_p_ is reduced from 3.0 µA to 2.0 µA for (*W/L*)_p_ =100 and from 1.0 µA to 600 nA for (*W/L*)_p_ =20. The net effect of the hysteresis is comparable to halving (*W/L*)_p_ from 100 to 50 and 20 to 10 (which in this case results in an even better match).

Fig. S4 I_p_/I_stat_ versus (W/L)_p_ for different V_DD_

**Gain plots**

In Fig. S3 the gain plots for the voltage transfer characteristics (VTC) of Fig. 3e,f,g,h are shown. The gain improves by changing (*W/L*)_p_ from 100 to 20. Note that the gain will be affected by the modelling (or measurement) resolution of *V*_in_, especially when there is a sharp transition. I.e. changing the resolution from 0.01V to 0.1V in Fig. S3d reduces the maximum gain at a supply voltage of 5V from 20 to about 10.

**
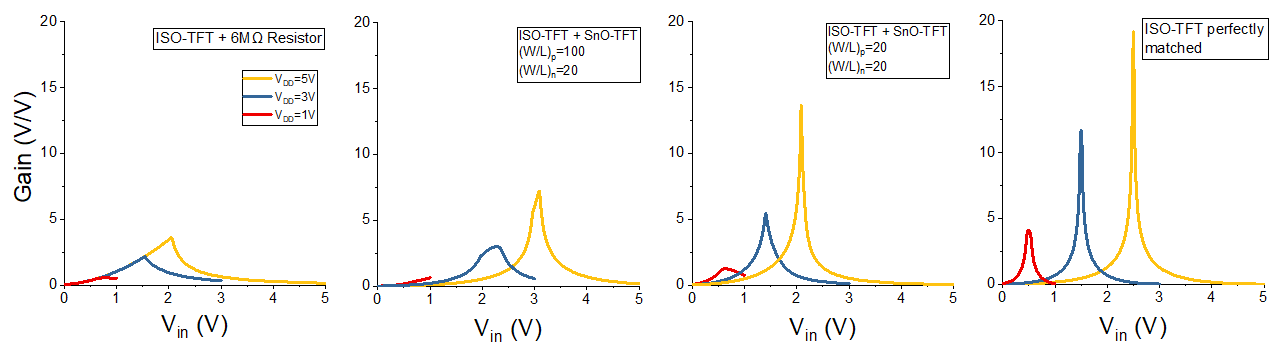
**

Fig. S3 Output characteristics of ISO TFT (W/L)_n_=20 (blue) with (a) 6MΩ resistor, (b) SnO (W/L)_p_=100, (c) SnO (W/L)_p_=20, (d) perfectly matched p-type TFT

**Plot of *I*_p_/ *I*_stat_ versus (*W/L*)_p_**

In Fig. S4 a plot is shown of *I*_p_/ *I*_stat_ versus (*W/L*)_p_ for different *V*_DD_. This shows that *I*_p_/ *I*_stat_ reaches its maximal value for the optimal (*W/L*)_p_=5. Note that at *V*_DD_ = 1V, the optimal (*W/L*)_p_ is slightly lower (1.7) as it is a function of *V*_DD_, but for 5V < *V*_DD_ < 10V, it the optimal (*W/L*)_p_ is ~ 5.

**Acknowledgements**

This work was supported by the UKRI Engineering and Physical Sciences Research Council through the Centre of Doctoral Training in Integrated Photonic and Electronics Systems (EP/L015455/1) and grant EP/P027032/1. For the purpose of open access, the author has applied a Creative Commons Attribution (CC BY) licence  to any Author Accepted Manuscript version arising.
